# Impact of Previous Physical Activity Levels on Symptomatology, Functionality, and Strength during an Acute Exacerbation in COPD Patients

**DOI:** 10.3390/healthcare6040139

**Published:** 2018-11-29

**Authors:** Laura López-López, Irene Torres-Sánchez, Ramón Romero-Fernández, María Granados-Santiago, Janet Rodríguez-Torres, Marie Carmen Valenza

**Affiliations:** Faculty of health of Sciences, University of Granada, Granada 18016, Spain; lauralopez@ugr.es (L.L.-L.); irenetorres@ugr.es (I.T.-S.); ramon-r-f@outlook.es (R.R.-F.); dulce.maria.1994.25@gmail.com (M.G.-S.) jeanette92@ugr.es (J.R.-T.)

**Keywords:** COPD, chronic obstructive pulmonary disease, physical activity

## Abstract

The main objective of this study is to determine the relationship between physical activity (PA) level prior to hospitalization and the pulmonary symptomatology, functionality, exercise capacity, and strength of acute exacerbated chronic obstructive pulmonary disease (COPD) patients. In this observational study, all data were taken during the patient’s first day in hospital. Patients were divided into two groups (a PA group, and a physical inactivity (PI) group), according to the PA level evaluated by the Baecke questionnaire. Cough status was evaluated by the Leicester Cough Questionnaire (LCQ), and dyspnea was assessed using the modified Medical Research Council dyspnea scale (mMRC). Functionality was measured by the Functional Independence Measure (FIM) and the London Chest Activity of Daily Living scale (LCADL). Exercise capacity was evaluated by the two-minute step-in-place (2MSP) test, and strength assessed by dynamometry. A total of 151 patients were included in this observational study. Patients in the PI group obtained worse results compared to the PA group, and significant differences (*p* < 0.05) were found in all of the variables. Those COPD patients who regularly perform PA have less dyspnea and cough, as well as better functionality, exercise capacity and strength during an exacerbation, without relationship to the severity of the pathology.

## 1. Introduction

Chronic obstructive pulmonary disease (COPD) is an excessive inflammatory response in the airway, which generates a persistent airflow limitation in response to chronic exposure to harmful particles or gases. The three main symptoms in these patients are cough, dyspnea, and sputum production [1]. The development of COPD is interspersed by exacerbations: Acute events, associated with an accelerated decline in lung function and health status [2], a reduction in quality of life, and an increase in morbidity and mortality [3]. Acute exacerbations of COPD are the main reason for hospitalization in these patients [4].

Despite the effect of COPD on the lungs, it has been characterized as a systemic disease due to the significant extrapulmonary manifestations that patients present [5]. Extrapulmonary symptoms include weakness of the skeletal musculature and emaciation, and have been associated with intolerance to exercise and deterioration in the state of health [6]. 

Typically, COPD patients are in a downward spiral of symptom-induced inactivity. Daily physical activity (PA) is reduced, even with the mildest forms of the disease [7]. Royo et al. [8] showed that a third of patients admitted to the pneumology department in Spain are sedentary. Pitta et al. [7] evidenced that most COPD patients, when compared with sedentary healthy subjects, spend less time standing and walking, and more time lying and sitting. 

A significant reduction in strength has been observed in both knees of these patients [9]. This is the main reason for the limitation in activities (such as climbing stairs or walking), and is a predictor of mortality in severe COPD patients [10].

For this reason, PA is the cornerstone of respiratory programs in COPD patients [11]. Some studies have evidenced a relationship between PA and the risk of mortality [12], lung function decline [13], or mental-health status [14].

Nevertheless, the relationship between PA level prior to hospital admission and the severity of pulmonary and extrapulmonary symptoms is not clear.

The objective of this study is to determine whether PA level in COPD patients prior to hospitalization is related to the pulmonary symptomatology, functionality, exercise capacity, and strength during exacerbation.

## 2. Materials and Methods

### 2.1. Participants 

In this observational study, severe COPD patients were recruited from the respiratory service of the San Cecilio and Virgen de las Nieves Hospitals in Granada, Spain. The study was carried out from September 2017 to June 2018. The inclusion criteria were: COPD patients (stages III and IV according to GOLD [14]), hospitalized due to an exacerbation, and older than 40 years of age. Patients were not included in the study if they had severe comorbidities (such as neurological disorders or orthopedic diseases) or cognitive impairment that would interfere with the evaluation, and those who did not sign the inform consent (annexed 1). 

The study was approved by the Ethical Committee of the San Cecilio and Virgen de las Nieves University Hospitals, Granada. All patients gave their written informed consent.

### 2.2. Measurements

All of the variables were measured during the patient’s first day of hospital admission, by the same physiotherapist. To better describe the sample, data on the history and current status of the disease, smoking history, and anthropometrical measures were collected. 

Spirometry was performed following the guidelines of the European Respiratory Society (ERS) and the American Thoracic Society (ATS) [15]. Comorbidity index was assessed by the Charlson comorbidity index, and quality of life was assessed by the Saint George Respiratory Questionnaire (SGRQ). 

The main outcomes were symptomatology, functionality, exercise capacity, and strength. Patients were divided into two groups, a PA group and a physical inactivity (PI) group, according to the cutoff point of the Spanish version of the modified Baecke physical activity questionnaire (which includes items about household, sport, and leisure time activities). The total score ranges between 0 and 47.56 points, and it indicates the degree of sedentary lifestyle. Patients with scores less than 9 points were included in the PI group, and patients with scores higher than 9 points were included in the PA group [16].

Pulmonary symptoms were evaluated using the modified Medical Research Council dyspnea scale (mMRC) to evaluate dyspnea, and the Leicester Cough Questionnaire (LCQ) to evaluate cough.

The mMRC dyspnea scale is a reliable and validated questionnaire that contains six items about perceived breathlessness. Each question ranges from 0 to 5, and a higher score indicates worse dyspnea [17].

The LCQ evaluates the relationship of cough and quality of life in the previous 24 h. The total score ranges from 3 to 21, with a higher score indicating better results [18].

Extrapulmonary symptoms include functionality, exercise capacity, and strength.

Functionality was measured using the London Chest Activity of Daily Living scale (LCADL) and the Functional Independence Measure (FIM).

The LCADL measures dyspnea limitation during exercise and activities of daily living in COPD patients. It includes 15 items (personal care, domestic activities, PA and leisure), with total score ranging from 0 to 5. Higher values indicate a higher level of functional impairment related to dyspnea [19].

The FIM evaluates physical and cognitive functionality with 18 items (13 motor and 5 cognitive), scored from 1 to 7. A higher score indicates a higher level of functionality [20].

Exercise capacity was measured using the two-minute step-in-place (2MSP) test [21]. The patients were asked to raise each knee, in turn, over two minutes. The score of this test is the number of times that the right knee arrives at the required height (a point between the patella and the iliac crest) in the two minutes.

Finally, lower and upper limb strength was measured by dynamometry. Lower-limb strength was evaluated in the quadriceps (dominant leg), using a portable handheld dynamometer [22] (Lafayette Manual Muscle Testing System, model 01163, Lafayette, IN, USA). 

Upper-limb strength was measured in the dominant hand, using a portable handheld dynamometer (TEC-60; Technical Products, USA.) [23]. 

In both cases, the highest value of three repetitions (allowing patients to rest between each measurement) was used in the statistical analysis.

### 2.3. Statistical Analysis

G Power 3.1.9.2. was used to calculate the sample size with the FIM. To detect an effect size of 0.5, with a desired power of 0.85 and an alpha of 0.05, the sample size should be at least 146 participants. Considering 10% dropout, a total of 151 patients were recruited for this study. 

Statistical analysis was performed using the SPSS statistical package, version 20.0. Normal distribution of the data was assessed with the Kolmogorov-Smirnov test (with Lilliefors correction). The homogeneity of variances was determined using Fisher’s F-test. If both conditions were satisfied, parametric tests were used (i.e.; Student’s *t*-test, paired Student’s *t*-test, and an ANOVA, followed by multiple comparisons using Tukey’s test, if necessary). If any of the conditions were not satisfied, a nonparametric test (i.e.; Mann-Whitney) was used instead. In all instances, α = 5%.

## 3. Results

**Figure 1:**
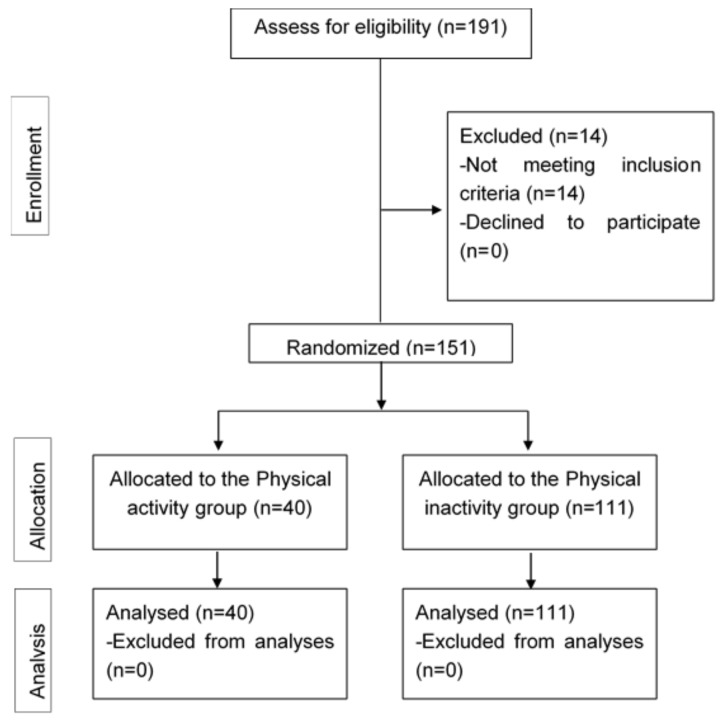
PRISMA flow chart of the participants.

The study flowchart is reported in Figure 1: 191 patients were screened, and 151 were actually recruited, randomized, and included in the analysis.

In Table 1, we see the baseline characteristics of the patients.

As we see in Table 1, the statistical analysis did not reveal the existence of statistical differences between the groups, except BMI (<0.001), Charlson comorbidity index (*p* = 0.019), and quality of life (<0.001). 

Table 2 shows the differences in the main outcomes of both groups.

As we see in Table 2, patients in the PI group obtained worse results compared to the patients in the PA group, and significant differences (*p* < 0.05) were found in all the variables.

## 4. Discussion

The objective of this study was to determine whether the PA level in COPD patients prior to hospitalization is related to the pulmonary symptomatology, functionality, exercise capacity, and strength during an exacerbation.

Our results have revealed that patients who regularly perform PA obtained less respiratory symptoms, and better extrapulmonary symptoms, on hospital admission due to an exacerbation. 

Physical inactivity is recognized as a significant risk factor in the exacerbation of COPD [24], and the relationship between daily PA and the reduction of hospital admissions has been studied by many authors [25,26]. Garcia-Aymerich et al. [27] revealed that patients with COPD who perform PA in their daily life have a substantially reduced risk of hospitalization due to exacerbation, without relationship to the severity of the pathology, nutritional status factors, or participation in respiratory rehabilitation. The reduced risk of hospitalization of COPD patients performing PA might explain the dissimilarity of sample size of our groups, and why there are more patients in the PI group.

The relationship between PA and the reduction in hospitalization due to acute exacerbation of COPD could be explained by exercise leading to a better conditioned cardiovascular system, which would adapt better to the increase in oxygen intake in respiratory muscles that occurs during COPD exacerbation [28]. 

Our results have shown better pulmonary and extrapulmonary symptoms in the PA group. 

### 4.1. Pulmonary Symptoms

It has been also shown [29] that a relationship exists between morning pulmonary symptoms and PA, without association to the disease stages. Patients with more symptomatic COPD performed significantly lower PA. Morning pulmonary symptoms were related to main clinical outcomes as increased anxiety and depression, lower health status, fewer steps a day, more symptoms, and less time in moderate and vigorous PA. This is in line with our results, which have shown that respiratory symptoms (dyspnea and cough) are related to physical activity (that is, the PA group of COPD patients showed less symptoms in an exacerbation). In the same line, Camillo et al. [29] showed a relationship between heart rate variability reduction and a lower level of PA in COPD patients. 

### 4.2. Extrapulmonary Symptoms

Camillo et al. also revealed that sedentary COPD patients obtained worse functional status, health-related quality of life, and respiratory and peripheral muscle force results without relation to the disease severity [29]. Additionally, the study of Iwakura et al. [30] revealed that deficits in balance are independently associated with PI. All these studies agreed with our results, which have shown that inactive COPD patients have worse functionality, exercise capacity, and strength.

Nevertheless, future clinical trials are needed to increase understanding of the relationship between PA and hospitalization due to exacerbation in COPD patients.

Some limitations should be explained: First, the difference in the sample size in the two groups can be considered as a limitation of this study. 

Second, the assessment of patients included self-reported measures. However, all of the questionnaires included in this study were validated, and have been previously used in COPD studies.

Finally, our results cannot be generalized to the non-hospital population, as patients were recruited during the hospitalization period due to an exacerbation of the disease.

## 5. Conclusions

We conclude that patients with COPD who regularly perform PA have less dyspnea and cough and better functionality, exercise capacity, and strength during an exacerbation.

## Figures and Tables

**Table 1:** Characteristics of patients at baseline.

Variables	PA Group (*n* = 40)	PI Group (*n* = 111)	*p*-Value
Age (years)	71.85 ± 7.65	71.52 ± 10.12	0.853
BMI (kg/m2)	31.73 ± 5.36	27.80 ± 5.13	<0.001 **
FVC predicted	50.52 ± 18.34	48.82 ± 20.46	0.662
FEV1 predicted	40.43 ± 17.50	34.66 ± 17.52	0.093
Hospital length of stay (days)	8.88 ± 1.72	9.72 ± 4.26	0.355
Charlson	5.88 ± 1.88	4.99 ± 1.83	0.019 *
SGRQ	51.40 ± 13.79	64.49 ± 12.39	<0.001 **

PA: Physical activity: PI: Physical inactivity; BMI: Body Mass Index; FVC: Force Vital Capacity; FEV1: Forced expiratory volume in the first second; Charlson: Charlson Comorbidity Index; SGRQ: Saint George Respiratory Questionnaire. Data are presented as mean values ± standard deviation. * *p* < 0.05, ** *p* < 0.001.

**Table 2:** Differences in symptomatology, functionality, exercise capacity and strength per group.

Variables	PA Group (*n* = 40)	PI Group (*n* = 111)	*p*-Value
Pulmonary symptoms	-	-	-
Dyspnea	-	-	-
mMRC	2.75 ± 0.98	3.36 ± 0.92	0.010 *
Cough	-	-	-
LCQ	16.47 ± 2.56	14.00 ± 3.71	0.002 *
Extrapulmonary symptoms	-	-	-
Functionality	-	-	-
LCADL	18.69 ± 6.61	29.69 ± 15.06	0.013 *
FIM	130.62 ± 5.91	111.63 ± 19.02	0.001 *
Exercise capacity	-	-	-
2MSP	43.33 ± 22.61	29.75 ± 23.44	0.016 *
Strength	-	-	-
Lower limb strength (Newton)	156.21 ± 75.01	103.97 ± 34.79	<0.001 **
Upper limb strength (Newton)	295.13 ± 68.01	227.74 ± 101.56	<0.001 **

PA: Physical activity: PI: Physical inactivity; mMRC: Modified Medical Research Council dyspnea scale; LCQ: Leicester Cough Questionnaire; LCADL: London Chest Activity of Living Scale; FIM: Functional Independence Measure; 2MSP: Two-minute step-in-place. Data are presented as mean values ± standard deviation. * *p* < 0.05, ** *p* < 0.001.
